# Aberrant fatty acid profile and FFAR4 signaling confer endocrine resistance in breast cancer

**DOI:** 10.1186/s13046-019-1040-3

**Published:** 2019-02-22

**Authors:** Xiao Chu, Qi Zhou, Yingchun Xu, Jingting Jiang, Qing Li, Qianjun Zhou, Qiong Wu, Min Jin, Hui Wang, Yuting Gu, Xue Wang, Bei Wang, Songbing He, Xiaozhou He, Changping Wu, Fengchun Zhang, Yanyun Zhang

**Affiliations:** 1 Department of Tumor Biological Treatment, The Third Affiliated Hospital of Soochow University, Institutes for Translational Medicine, Soochow University, Changzhou, Jiangsu China; 2 Key Laboratory of Tissue Microenvironment and Tumor, Shanghai Institutes for Biological Sciences, Chinese Academy of Sciences, Shanghai, China; 3 Department of Oncology, Renji Hospital, Shanghai Jiao Tong University School of Medicine (SJTUSM), Shanghai, China; 4 Department of Oncology, Suzhou Kowloon Hospital and Shanghai Ruijin Hospital, SJTUSM, Suzhou, Jiangsu China; 5 Department of Radiation Oncology, Fudan University Shanghai Cancer Center, Fudan University, Shanghai, China

**Keywords:** Fatty acid receptor, FFAR4, Hormone receptor-positive breast cancer, Fatty acids, Gas chromatography-mass spectrometry, Tumor microenvironment, Biomarker, Endocrine resistance

## Abstract

**Background:**

Evidence suggests that fatty acid receptor FFAR4 plays a tumor-promoting role in adipose tissue-adjacent malignancies, but its clinical relevance remains unexplored. Here, we investigated the clinical significance and underlying mechanisms of FFAR4 in hormone receptor-positive breast cancer (HRPBC).

**Methods:**

FFAR4 expression was assessed by immunohistochemistry in an exploration cohort of 307 breast cancer cases collected from two independent institutes. Two public breast cancer microarray datasets served as validation cohorts. Gas chromatography-mass spectrometry was employed to identify FFAR4 ligands in normal and cancerous breast tissues. Survival analyses were performed in all cohorts and designated molecular subgroups. Mechanistic studies were performed in vitro in hormone receptor-positive breast cancer cell lines MCF-7 and T-47D.

**Results:**

Aberrant FFAR4 expression and endogenous FFAR4 ligands were identified in breast cancer tissues, five FFAR4 ligands showed significantly elevated proportions in cancerous versus normal tissues. In the exploration cohort, FFAR4 was demonstrated as an independent prognostic factor for recurrences (HR: 2.183, 95% CI: 1.360–3.504, *P* = 0.001) and breast cancer-specific deaths (HR: 2.102, 95% CI: 1.173–3.766, *P* = 0.013) in HRPBC cases. In contrast, FFAR4 expression was not associated with prognosis in hormone receptor-negative cases. In the validation cohorts, FFAR4 mRNA levels were also observed to be associated with disease recurrence in estrogen receptor-positive cases, but not so in estrogen receptor-negative cases. FFAR4 activation by endogenous ligands and a synthetic ligand TUG891 significantly dampened tamoxifen’s efficacy on HRPBC cells, whereas FFAR4 knockdown or antagonist AH7614 abrogated this effect. Furthermore, FFAR4-induced tamoxifen resistance was dependent on ERK and AKT pathways in HRPBC.

**Conclusions:**

Our results establish a novel role of FFAR4 and its ligands in the complicated interactions between tissue lipid profile and cancer biology. FFAR4 signaling confers tamoxifen resistance in HRPBC cell line and FFAR4 expression can serve as a prognostic biomarker for tamoxifen-treated HRPBC patients. FFAR4 may serve as a potential target for anti-breast cancer therapies, especially in endocrine resistant cases.

**Electronic supplementary material:**

The online version of this article (10.1186/s13046-019-1040-3) contains supplementary material, which is available to authorized users.

## Introduction

Breast cancer is the most prevalent malignancy in women, and its incidence has been rising along with rapid urbanization and adoption of modern life style in developing countries like China [1]. Despite significant progresses in breast cancer treatment during the past few decades, a substantial proportion of breast cancer survivors eventually succumb to this malignancy due to disease recurrence. This situation calls for more accurate prognostic parameters and better targeted and personalized therapies.

The tumor microenvironment is crucial for tumor development and progression. In breast cancer, due to its proximity to adipose tissues, the microenvironment is enriched with adipocytes, which have been identified as an important component for disease progression. The cancer cells induce phenotype transformation and lipolysis of adipocytes, while adipocytes secret proinflammatory cytokines, proteases, and hormones to support cancer progression, and release bulk amounts of free fatty acids from lipolysis [2–4]. Fatty acids were previously regarded as energy source and building blocks to sustain the abnormally rapid proliferation of cancer cells, and enhanced lipogenic capacity was noticed in most human malignancies including breast cancer [5–8]. However, whether fatty acids per se act as tumor promoting signals remains elusive, partially due to the lack of knowledge in fatty acid-specific sensors/receptors. The recent deorphanization of several fatty acid receptors changed this situation [9, 10].

In particular, free fatty acid receptor 4 (FFAR4), also known as GPR120 and a receptor for long-chain free fatty acids, gained our attention. FFAR4 regulates various physiological processes including food preference [11, 12], secretion of several gastroenteropancreatic hormones [10, 13], adipogenesis [14], macrophage phenotype transformation and adipose inflammation [15, 16]. Importantly, FFAR4 signaling was shown to exert pro-survival and anti-apoptotic properties [17, 18]. Since cancer cells are known to “hijack” physiological functions of G protein-coupled receptors to serve their own purposes [19], we investigated whether cancer cells also exploit FFAR4 signaling. Our group have confirmed that FFAR4 signaling promoted angiogenesis and induced epithelial-mesenchymal transition [20]. During previous experiments, we found that FFAR4 was also expressed in human breast cancer tissues. Meanwhile, many long-chain fatty acids were detected in breast tissues, including FFAR4 ligands such as docosahexaenoic acid (DHA) [21–23]. However, these studies did not consider the fatty acids as stimulating signals, and have only taken normal breast tissues into account, further investigations into breast cancer tissues are needed. The evidence indicated that FFAR4 might be involved in breast cancer biology, but whether FFAR4 affects breast cancer in clinical settings remains enigmatic.

Based on the accumulating evidence of FFAR4 and its ligands in breast tissues, we hypothesized that breast cancer cells may utilize fatty acids in situ as stimulating signals via expressing FFAR4 and therefore exert detrimental effects on disease outcome. In this study, we validated our hypothesis and showed that FFAR4 expression can serve as a prognostic marker in tamoxifen-treated hormone receptor-positive breast cancer (HRPBC) patients. Furthermore, we identified FFAR4 ligands with elevated proportions in cancerous versus normal breast tissues. Activation of FFAR4 by these endogenous ligands or synthetic agonist TUG891 conferred tamoxifen resistance in HRPBC cells, whereas FFAR4 knockdown or antagonist AH7614 abrogated this effect. FFAR4-induced tamoxifen resistance is dependent on ERK and AKT pathways. Altogether, our results demonstrate FFAR4 as a prognostic biomarker in HRPBC patients and as a potential target for breast cancer therapies, especially in endocrine resistant cases.

## Materials and methods

### Exploration cohort

The exploration cohort was selected from all patients who received curative breast cancer surgery between 2003 and 2009 at Renji Hospital, Shanghai Jiao Tong University School of Medicine, Shanghai, China or the Third Affiliated Hospital of Soochow University, Changzhou, Jiangsu, China. Briefly, only female patients diagnosed with American Joint Committee on Cancer stage I to III breast cancer were picked for pathological evaluation. Cases with sufficient archived tissues and pathological information were selected for follow-ups, 307 patients were traced in the follow-up constituted the exploration cohort. The patient selection workflow was visualized in Additional file 1: Figure S1. Cohort clinical characteristics were summarized in Table 1.

**Table 1:** Patients’ characteristics according to FFAR4 status

Characteristics	All patients				Hormone receptor-positive patients			
	Total (*n* = 307) (%)	FFAR4 Low (*n* = 158) (%)	FFAR4 High (*n* = 149) (%)	*P*	Total (*n* = 212) (%)	FFAR4 Low (*n* = 99) (%)	FFAR4 High (*n* = 113) (%)	*P*
Age at diagnosis				0.915				0.753
≤ 35	10 (3)	6 (60)	4 (40)		10 (5)	6 (60)	4 (40)	
36–50	116 (38)	60 (52)	56 (48)		82 (39)	40 (49)	42 (51)	
51–64	123 (40)	64 (52)	59 (48)		74 (35)	32 (43)	42 (57)	
≥ 65	58 (19)	28 (48)	30 (52)		46 (21)	21 (46)	25 (54)	
Menstrual status				0.909				0.383
Premenopausal	131 (43)	68 (52)	63 (48)		93 (44)	45 (48)	48 (52)	
Postmenopausal	176 (57)	90 (51)	86 (49)		119 (56)	54 (45)	65 (55)	
AJCC stage			0.989					0.837
I	85 (28)	43 (50)	42 (50)		64 (30)	30 (47)	34 (53)	
II	140 (45)	73 (52)	67 (48)		94 (44)	42 (45)	52 (55)	
III	82 (27)	42 (51)	40 (49)		54 (26)	27 (50)	27 (50)	
Histology				0.694				0.915
Ductal	270 (88)	139 (51)	131 (49)		182 (86)	84 (46)	98 (54)	
Lobular	15 (5)	9 (60)	6 (40)		13 (6)	7 (54)	6 (46)	
Other	22 (7)	10 (45)	12 (55)		17 (8)	8 (47)	9 (53)	
Grade				0.199				0.087
1 or 2	187 (61)	102 (55)	85 (45)		137 (65)	70 (51)	67 (49)	
3	120 (39)	56 (47)	64 (53)		75 (35)	29 (39)	46 (61)	
Tumor size, cm				0.416				0.890
≤ 2	124 (40)	60 (48)	64 (52)		94 (44)	43 (46)	51 (54)	
> 2	183 (60)	98 (54)	85 (46)		118 (56)	56 (47)	62 (53)	
Lymph node status				0.909				0.491
Positive	142 (46)	74 (52)	68 (48)		95 (45)	47 (50)	48 (50)	
Negative	165 (54)	84 (51)	81 (49)		117 (55)	52 (44)	65 (56)	
ER				0.023*				1.000
Positive	198 (64)	92 (46)	106 (54)		198 (93)	92 (46)	106 (54)	
Negative	109 (36)	66 (61)	43 (39)		14 (7)	7 (50)	7 (50)	
PR				0.250				0.267
Positive	177 (58)	86 (49)	91 (51)		177 (83)	86 (49)	91 (51)	
Negative	130 (42)	72 (55)	58 (45)		35 (17)	13 (37)	22 (63)	
HER				0.901				1.000
Positive	92 (30)	48 (52)	44 (48)		51 (24)	24 (47)	27 (53)	
Negative	215 (70)	110 (51)	105 (49)		161 (76)	75 (47)	86 (53)	
Ki-67				0.171				0.008*
≤ 20%	157 (51)	87 (55)	70 (45)		123 (58)	67 (54)	56 (46)	
> 20%	150 (49)	71 (47)	79 (53)		89 (42)	32 (36)	57 (64)	
Molecular subtype				0.066				0.279
Luminal A	75 (24)	40 (53)	35 (47)		75 (35)	40 (53)	35 (47)	
Luminal B HER2-	86 (28)	35 (41)	51 (59)		86 (41)	35 (41)	51 (59)	
Luminal B HER2+	51 (17)	24 (47)	27 (53)		51 (24)	24 (47)	27 (53)	
HER2 non-luminal	41 (13)	25 (61)	16 (39)					
Hormone receptor				0.014*				
Positive	212 (69)	99 (47)	113 (53)					
Negative	95 (31)	59 (62)	36 (38)					

Abbrevation: *ER* estrogen receptor, *PR* progesterone receptor, *HER2* human epidermal growth factor receptor 2; hormone receptor positive = ER or PR positive. Fisher’s exact test were used to compare the distribution of clinical features between FFAR4 low and high patients. (*) Statistically significant

Formalin-fixed and paraffin-embedded (FFPE) tissues were assessed by expert pathologists, 4 μm full-face sections were procured for immunohistochemistry (IHC). Archived sections for estrogen receptor (ER), progesterone receptor (PR), human epidermal growth factor receptor 2 (HER2), and Ki67 status were evaluated for breast cancer subtype identification based on pathological subtype definitions, St Gallen consensus [24]. All HRPBC patients were subjected to adjuvant endocrine therapy (tamoxifen).

Informed consent for the use of resected tissues was obtained from all patients. This study was approved by the independent ethics committees of each institute and was conducted in accordance with the Helsinki Declaration. Reporting Recommendations for Tumor Marker Prognostic Studies (REMARK) criteria were followed in this study [25].

### Follow-up and event definition

The median follow-up for the whole cohort was 83 months from surgery (range, 5 to 140 months). Events of interest including death, recurrence and secondary tumors were collected during follow-ups with additional verification via cross-referencing medical records. Vital status and causes of death were double-confirmed at local population registries.

The primary and secondary measures of FFAR4 prognostic value were the events of disease recurrence and breast cancer-specific death, respectively. For recurrence-free survival (RFS), recurrence of local or regional disease, distant recurrence and death from breast cancer were considered events, survival time was censored at deaths due to other causes and at the onset of contralateral breast cancer. For breast cancer-specific survival (BCSS), only death from breast cancer was considered an event, survival time was censored at deaths due to other causes.

### IHC assay and result evaluation

IHC staining was performed on full-face tumor sections as described previously [20, 26]. Briefly, after deparaffinization and antigen retrieval, sections from FFPE tissue blocks were incubated with a primary FFAR4 antibody (Abcam, Cambridge, UK, ab97272, 1:500 dilution), visualized with non-biotin detection system (GTVision III Detection System, Gene Tech, Shanghai, China, GK500710), counterstained with hematoxylin, dehydrated in graded alcohols, cleared in xylene, and coverslipped.

The percentage of positive cells and the staining intensity of FFAR4 were scored by two expert pathologists blinded to patient outcomes using H-score system [27–31]. The staining intensity of tumor cells was scored into four categories: negative (0), weak (+, light brown staining, visible only with high magnification), moderate (++, between + and +++), and intense (+++, visible with low magnification, dark brown staining). A 10-tiered scale (10 to 100%) was used to score the percentage of FFAR4 positive tumor cells. The H-score was calculated with the following formula: 1 × (percentage of cells staining weakly [+]) + 2 × (percentage of cells staining moderately [++]) + 3 × (percentage of cells staining intensely [+++]) and the overall score ranged from 0 to 300. Only membranous/cytoplasmic staining in tumor cells was considered, staining in macrophages and adipocytes was not counted in scoring. Tumors with H-score >150 were considered FFAR4 high, optimal cutoff determination is described in the “statistical analysis” section.

### Validating cohorts

EBI ArrayExpress dataset E-MTAB-365 and Gene Expression Omnibus dataset GSE4922 were employed as validation cohorts. Sample selection in the validation cohorts was visualized in Additional file 1: Figure S1. The raw data and supplementary clinical information were downloaded, and gene expression data were MAS5 normalized and log-transformed in the R statistical environment (www.r-project.org) using Affy Bioconductor library. FFAR4 mRNA levels (range 0.7286 to 7.4392 in E-MTAB-365 and 2.109 to 7.766 in GSE4922) for all cases with both estrogen receptor status and survival information were extracted and used for validating analysis. The best performing thresholds (4.779 for E-MTAB-365 and 6.004 for GSE4922) were selected as a cut-off value for event visualization, optimal cutoff determination is described in the “statistical analysis” section. FFAR4 expression were analyzed as continuous or binary variable in multivariable analysis.

### Fatty acid extraction, identification and quantification

Gas chromatography-coupled mass spectrometry (GC-MS) was employed for fatty acid quantification and identification in normal and cancerous breast tissues of 19 breast cancer patients (3 luminal A, 5 luminal B HER2-, 2 luminal B HER2+, 3 HER+ non-luminal and 6 triple-negative). Tissues were kept in liquid nitrogen after resection.

For fatty acid extraction, frozen tissues were crushed into fine powder in liquid nitrogen, approximately 0.1 g of the powder was transferred to a screw-capped glass tube containing 1 mL sulfuric acid and methanol mixture (5% *v*/v), and 10 μl methyl nonadecanoate (5.56 mg/mL) was added as the internal standard. Then the tubes were blown with nitrogen to drive out air, sealed immediately and heated at 80 °C for 90 min, then cooled in fridge for 10 min. 1 mL distilled water and 500 μL hexane were then added to each tube. The tubes were vortexed for 3 min, then centrifuged at 3000 r.p.m. for 10 min. The upper phase (hexane phase) containing fatty acid methyl esters (FAME), was taken for GC-MS analysis.

A 7890A Gas Chromatograph coupled with a 5975C Mass Spectrometer (Agilent, Santa Clara, CA) was used for FAME identification. DB-5 ms capillary column (30 m × 0.25 mm × 0.2 μm; Agilent) was used to separate compounds; Sample injections (1 μL) were performed in splitless mode with the injection port temperature at 270 °C. Helium was used as carrier gas at a constant flow of 1 mL/min. The capillary column temperature was initially set at 70 °C for 5 min, then increased to 200 °C at 25 °C/min, and then to 280 °C at 2 °C /min. The total run time was 50.2 min. The operating temperatures for quadruple, ion source and transfer line were set at 150, 230 and 280 °C, respectively. Electron impact ionization mass spectra results were recorded with the ionization voltage set at 70 eV and electron multiplier voltage at 1765 V. Mass spectra were scanned from 33 to 500 amu in total ion chromatogram (TIC) mode. Prepared samples were measured under identical conditions by the same technicians in a random sequence.

FAME were identified using a MSD ChemStation software (Agilent, version E.02.02.1431), FAME peak retention times and mass spectra were compared with those of reference compounds: a 37-component FAME Mix (Nu-chekprep, USA), a 26-component Bacterial Acid Methyl Ester (BAME) Mix (Supelco, USA), and a C18:1n7 FAME standard (Nu-chekprep, USA); when no standard reference was available, identification was carried out by referring the mass spectra to the 2011 NIST (National Institute of Standards and Technology) mass spectral library. 

### Cell culture and treatment

Human breast cancer cell lines MCF-7 and T-47D were obtained from American Type Culture Collection (ATCC, Manassas, VA, USA). Cells were maintained in RPMI 1640 supplemented with 10% FBS and 1% penicillin-streptomycin. To minimize disturbance of FFAR4 ligands in serum, and estrogen-like effect of phenol red, phenol red-free RPMI 1640 (Sigma-Aldrich, St. Louis, MO, USA) were supplemented with 10% heat-inactivated charcoal-stripped fetal bovine serum (SH30068, HyClone Laboratories, Logan, Utah). Since charcoal-stripping depletes estrogen from serum as well, 1 nM β-estradiol (Sigma-Aldrich) was resupplemented in the medium to provide stable estrogen concentration. Cells were cultured in this medium for 48 h before and during any treatment.

### Cell line transfection and transduction

FFAR4 knockdown and control MCF-7 cell lines were established as previously described [20]. Briefly, lentiviral vectors expressing FFAR4 shRNA or scrambled shRNA under U6 promoter were employed for FFAR4 knockdown according to manufacturer’s protocols. Cells resistant to puromycin were selected and passaged for further studies.

### Cell viability assays

Fatty acids and active tamoxifen metabolite 4-hydroxytamoxifen (4OHT) were obtained from Sigma and dissolved in ethanol. FFAR4-selective agonist TUG891 (Axon Medchem, Netherland) and antagonist AH7614 (Tocris, Minneapolis, MN, USA), ERK1/2 inhibitor SCH772984 and AKT inhibitor MK2206 (Selleck chemicals, Houston, TX, USA) were reconstituted in dimethyl sulfoxide. For cell viability assays, cells were seeded in 96-well plates and allowed to grow for 48 h, then treated with indicated time and reagents, inhibitors were added 30 min prior to other reagents. WST-1 substrate (Beyotime, Shanghai, China) was then added into culture medium, after 2 h incubation, spectral absorbance was measured at 450 nm for cell viability calculation.

### Immunoblotting

Immunoblotting was carried out as previously described [20]. ERK1/2, AKT, phospho-ERK1/2, phospho-AKT antibodies (Cell Signaling Technology, Danvers, MA, USA) and FFAR4 antibody (Abcam, 1:1000) were diluted according to manufacturers’ recommendations. All figures illustrating immunoblotting analyses are representative of at least three independent experiments.

### Statistical analysis

Associations between FFAR4 expression and clinical characteristics were evaluated via Fisher’s exact test. For cohort dichotomization, the optimal cutoff value was determined by calculating predictive accuracy of the different FFAR4 expression (H-score or mRNA level) for 5-year RFS, in the R statistical environment using the “survivalROC” package [32]. Kaplan and Meier plot was used to visualize the event-time distributions in different FFAR4 expression groups [33], and differences in recurrence and breast cancer-specific death were compared using the log-rank test [34]. Cox proportional-hazards regression analysis was used to evaluate the prognostic significance of FFAR4 H-score and mRNA levels as binary or continuous variables [35]. To compare fatty acid profiles between normal and cancerous tissues, the paired differences between tissues in each fatty acid was analyzed by Shapiro-Wilk test of normality; if the distribution was normal, paired samples T test was employed, in cases the distribution was not normal, Wilcoxon signed rank test was used. One-way ANOVA was employed to examine the effect of different concentrations of FFAR4 ligands on tamoxifen efficacy. Independent samples T test was used to determine the effects of FFAR4 knockdown and inhibitors.

All statistical tests were two-sided, and *P* values of less than 0.05 were considered statistically significant; data were shown as mean ± standard deviation. SPSS software, version 20 (IBM, Armonk, NY) was used for all statistical analyses. Kaplan-Meier plots were generated using GraphPad Prism 6 (GraphPad Software, San Diego, CA, USA).

## Results

### Aberrant FFAR4 expression in breast cancer tissues and its association with clinical characteristics

**Fig. 1:**
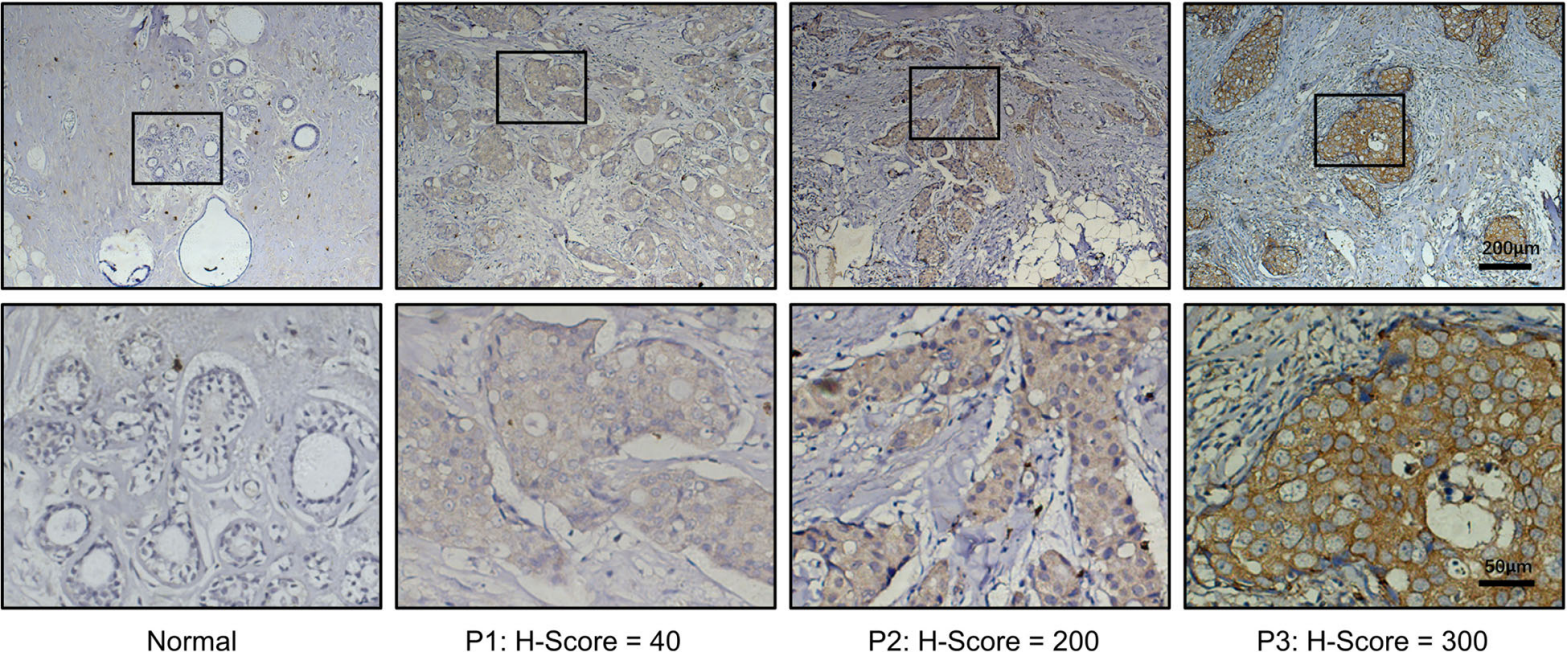
Differential FFAR4 expression in normal and cancerous human breast tissues. Representative examples of IHC staining and H-scores for FFAR4 in human breast tissues. Normal breast tissue: negative staining in normal breast epithelial cells. P1: Patient 1, H-score 40 = 1 x (40% cells staining weakly [1+]) + 2 x (0% cells staining moderately [2+]) + 3 x (0% cells staining strongly [3+]). P2: Patient 2, H-score 200 = 0 x (0% cells staining weakly [1+]) + 2 x (100% cells staining moderately [2+]) + 3 x (0% cells staining strongly [3+]). P3: Patient 3, H-score 300 = 1 x (0% cells staining weakly [1+]) + 2 x (0% cells staining moderately [2+]) + 3 x (100% cells staining strongly [3+]). Scale bar, 200 μm (top panel) and 50 μm (bottom panel)

Since FFAR4 expression in human breast tissue had never been reported before, we first investigated FFAR4 status in FFPE cancerous and normal breast tissues via IHC. As shown in Fig. 1, FFAR4 exhibited membranous and cytoplasmic localization and was differentially expressed in breast cancer cells while remaining negative in normal breast epithelial cells. Patients were classified FFAR4-high or FFAR4-low based on immunohistochemical H-score, and clinical characteristics were evaluated with respect to FFAR4 expression. In the exploration cohort, higher FFAR4 H-score was significantly associated with positive ER status (*P* = 0.023) and positive hormone receptor status (ER or PR positive, *P* = 0.014) but not with PR alone or other clinical characteristics (Table 1).

### FFAR4 ligands distribution in normal and cancerous breast tissues

**Fig. 2:**
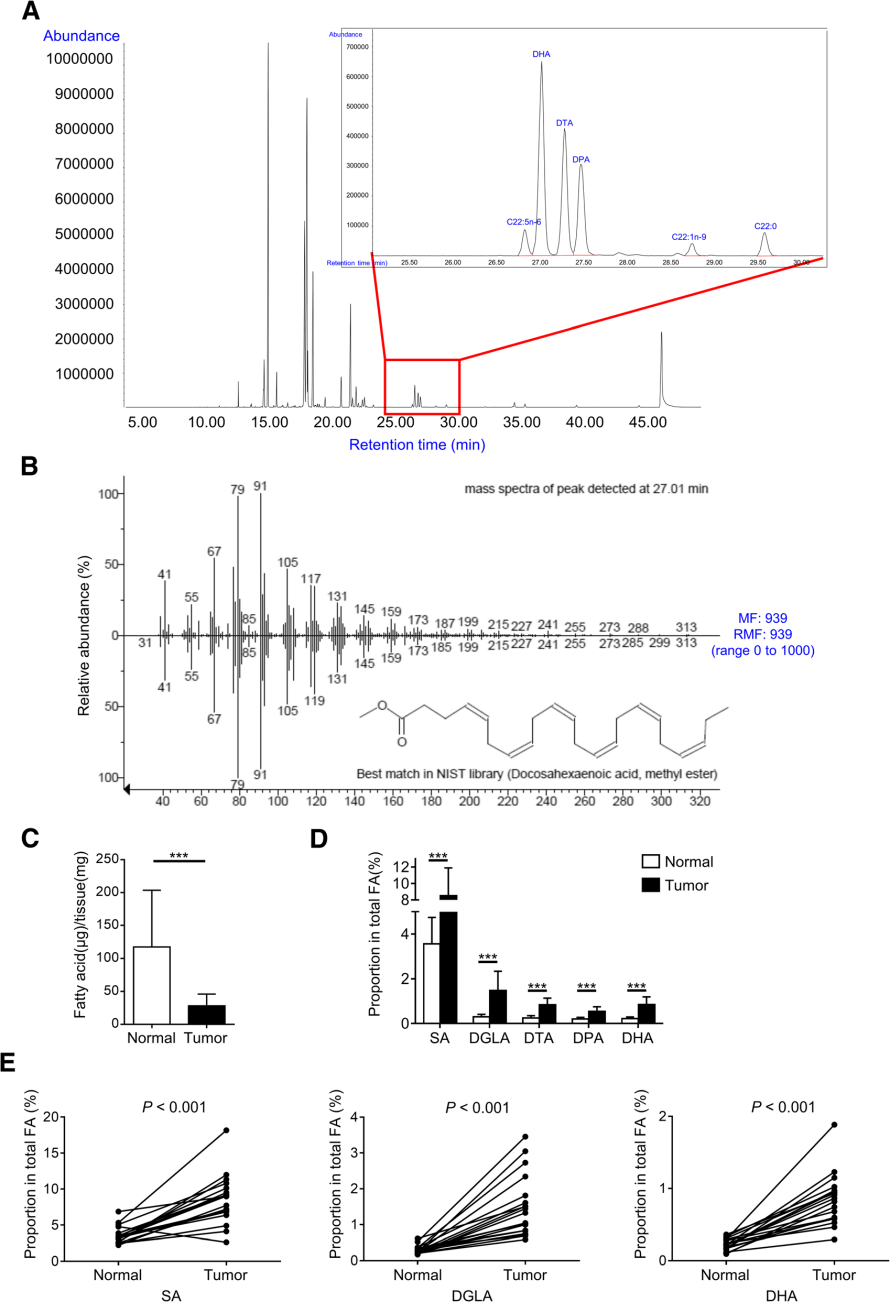
Endogenous FFAR4 ligand distribution in human breast tissues. **a** Representative gas chromatogram of breast cancer tissue samples. **b** Mass spectra of the FAME captured at retention time 27.010 min (top) shows excellent match (MF and RMF at 939 on a scale of 0 [no match] to 1000 [perfect match]) with the mass spectra of DHA methyl ester in NIST library (bottom). **c** Total fatty acid amount is significantly lower in cancerous compared to normal breast tissues (*n* = 19). **d** five FFAR4 ligands exhibited elevated proportions in cancerous compared to normal breast tissues (*n* = 19). **e** Detailed view of uniformly elevated proportions of SA, DGLA and DHA in cancerous compared to normal tissues (*n* = 19). Each line represents an individual subject. Bars represent mean ± S.D. *** *P* <  0.001. FAME: fatty acid methyl ester; MF: matching factor; RMF: reverse matching factor

The interaction of FFAR4 with its endogenous ligands is fundamental for its biological functions, therefore it is crucial to examine FFAR4 ligand abundance and distribution in breast cancer tissues. Here we employed GC-MS to analyze fatty acid profiles in both normal and cancerous breast tissues of 19 untreated breast cancer patients. Representative gas chromatogram and mass spectra of breast cancer tissues are illustrated in Fig. 2a and b, the complete fatty acid profiles were listed in Additional file 2: Tables S1 and S2. 24 fatty acids were identified in breast tissues, 12 of which were confirmed FFAR4 ligands [10, 36].

We also compared the fatty acid profiles between normal and cancerous breast tissues. To eliminate disturbance from individual differences (e.g. different nutritional status), the breast cancer tissue was paired with adjacent normal breast tissue from the same patient (matched samples). The total fatty acid quantities were significantly lower in cancerous breast tissues (28.03 ± 17.96 μg/mg) than in normal breast tissues (117.2 ± 86.27 μg/mg) (Fig. 2c, Additional file 2: Table S1).

Normal and cancerous breast tissues are composed of different cell types and may have different fatty acid profiles, therefore we compared fatty acid profiles as a proportion of total fatty acids in each tissue. Despite the dramatic drop in total fatty acid quantity, we observed elevated proportions of 5 FFAR4 ligands in cancerous breast tissues compared to adjacent normal breast tissues, namely C18:0 (Stearic acid, SA), C20:3n-6 (Dihomo-gamma-linolenic acid, DGLA), C22:4n-6 (Docosatetraenoic acid, DTA), C22:5n-3 (Docosapentaenoic acid, DPA) and C22:6n-3 (Docosahexaenoic acid, DHA) (Fig. 2d, Additional file 2: Table S2). SA, DGLA and DHA, classified as saturated, ω-6 polyunsaturated and ω-3 polyunsaturated FFAR4 ligands respectively, showed uniformly elevated proportions across individuals in tumor compared to normal breast tissues (Fig. 2e).

Since FFAR4 expression is correlated with hormone receptor status (Table 1), we classified the patients in GC-MS analysis into hormone receptor-positive (*n* = 10) or hormone receptor-negative (*n* = 9) subtypes and compared fatty acid profiles between cancerous and normal breast tissues in each subtype. The FFAR4 ligands with unabated quantities and elevated proportions remained the same regardless of hormone receptor status (Additional file 2: Tables S1 and S2), indicating that the FFAR4 ligands in situ are not likely regulated by hormone receptors in breast cancer tissues.

These results revealed that certain FFAR4 ligands are abundantly present and enriched in breast cancer tissues, which may trigger FFAR4 signaling and culminate in worse outcome for breast cancer patients.

### FFAR4 is an independent prognostic marker for recurrence and survival in tamoxifen-treated HRPBC patients

**Fig. 3:**
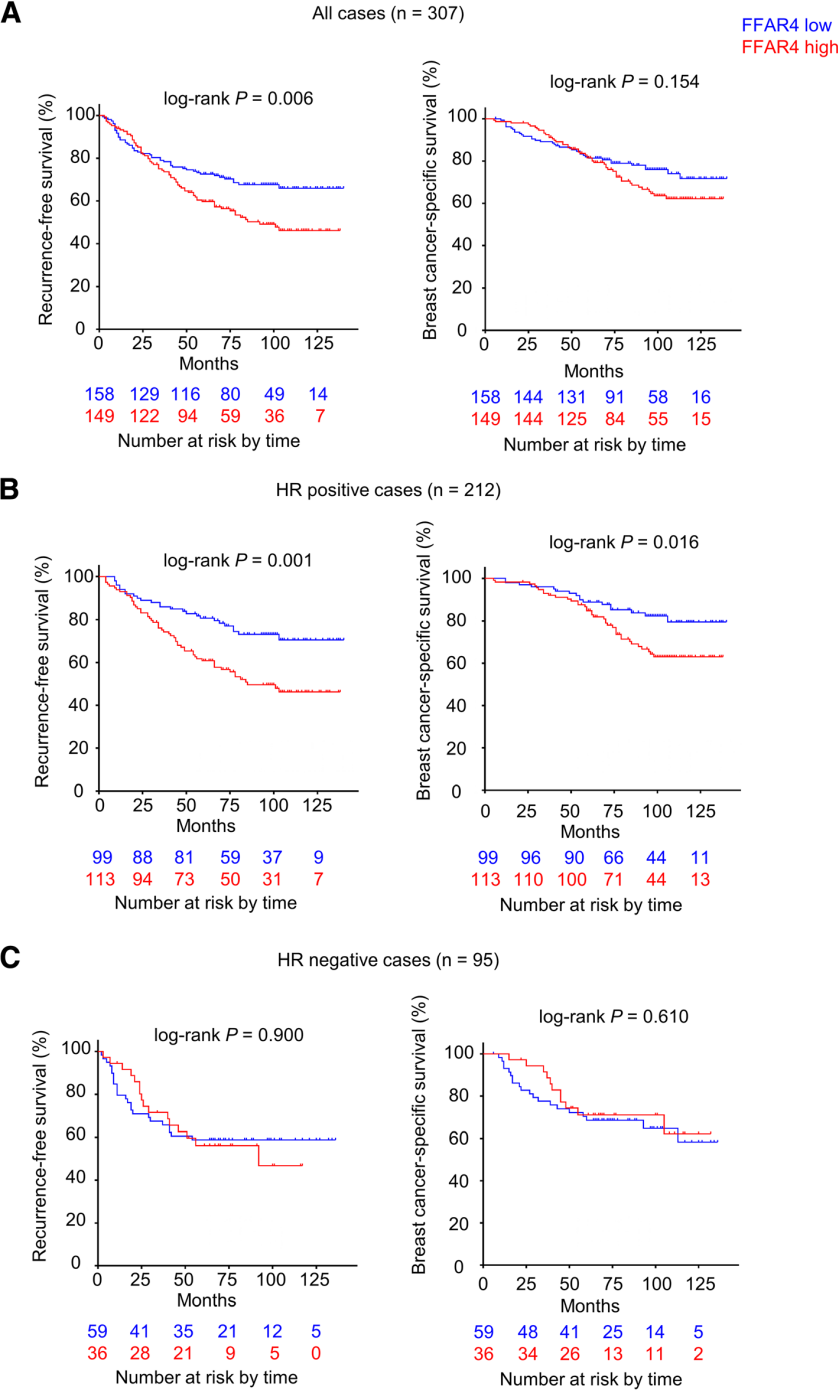
FFAR4 predicts outcome in tamoxifen-treated hormone receptor-positive breast cancer (HRPBC) patients. Kaplan-Meier curves of recurrence and breast cancer-specific deaths based on FFAR4 expression in the exploration cohort. RFS and BCSS of (**a**) the entire cohort (*n* = 307), (**b**) the HRPBC cohort (*n* = 212), (**c**) the hormone receptor-negative cohort (*n* = 95). Bottom numbers indicate patients at risk at different time points

With FFAR4/ligand presence in breast cancer tissues confirmed, we next compared RFS and BCSS between FFAR4-high and FFAR4-low patients in the exploration cohort (*n* = 307) collected from 2 independent institutions. At the time of analysis, 122 patients (39.7%) developed disease recurrences, and 85 patients (27.7%) died from breast cancer. As hypothesized, FFAR4-high patients had significantly worse RFS compared to FFAR4-low patients (Fig. 3a) (*P* = 0.006), but the BCSS difference between FFAR4-high and FFAR4-low groups had not reached statistical significance. (Fig. 3a) (*P* = 0.154).

It is well acknowledged that breast cancers are constituted by heterogeneous subtypes that differ substantially in intrinsic mechanisms and prognosis [24, 37–40]. To examine whether the prognostic value of FFAR4 expression is different between breast cancer subtypes, patients were classified into Luminal A (*n* = 75), Luminal B HER2 negative (*n* = 86), Luminal B HER2 positive (*n* = 51), HER2 positive non-luminal (*n* = 41) and Triple negative (*n* = 54) subtypes based on pathological subtype definitions [24]. In Luminal A, Luminal B HER2 negative and Luminal B HER2 positive sub-cohorts, higher FFAR4 expression showed tendency to be associated with worse prognosis (Additional file 1: Figure S2A-F). Meanwhile, no significant association was observed between FFAR4 and prognosis in HER2 positive non-luminal and Triple negative sub-cohorts (Additional file 1: Figure S2G-J).

The distinguishing results from different breast cancer subtypes reveals that the prognostic value of FFAR4 might only apply to HRPBC patients (Luminal A, Luminal B HER2 negative and Luminal B HER2 positive). Hence, we next compared survival between different FFAR4 groups in all HRPBC patients (*n* = 212, median follow-up 88 months), clinical characteristics summarized in Table 1, who were subjected to adjuvant endocrine therapy (tamoxifen).

At the time of analysis, 82 patients (38.7%) developed disease recurrences, and 54 patients (25.5%) died from breast cancer. As speculated, FFAR4-high patients in the HRPBC cohort showed significantly worse RFS (10-years RFS; FFAR4-low vs. high: 70.5% vs. 46.2%; *P* = 0.001; Fig. 3b) and BCSS (10-years BCSS; FFAR4-low vs. high: 79.5% vs. 63.0%; *P* = 0.016; Fig. 3b). In multivariable analysis, FFAR4 H-score retained prognostic significance for both RFS (binary, hazard ratio [HR]: 2.183, 95% confidence interval [CI]: 1.360–3.504, *P* = 0.001; continuous, HR: 1.058, 95% CI: 1.029–1.088, *P* < 0.001) and BCSS (binary, HR: 2.102, 95% CI: 1.173–3.766, *P* = 0.013; continuous, HR: 1.052, 95% CI: 1.018–1.088, *P* = 0.003;) independent of other clinical characteristics (Table 2). In stark contrast, FFAR4 status was not associated with RFS or BCSS in hormone receptor-negative patients (Fig. 3c).

**Table 2:** Multivariable analyses in the exploration HRPBC cohort

Covariates	Recurrence-free survival		Breast cancer-specific survival	
	HR (95% CI)	*P*	HR (95% CI)	*P*
FFAR4 (continuous)	**1.058 (1.029–1.088)**	**< 0.001**	**1.052 (1.018–1.088)**	**0.003**
Age at diagnosis	0.988 (0.968–1.009)	0.260	0.999 (0.974–1.024)	0.907
Grade (3 vs 2 or 1)	1.684 (1.068–2.655)	0.025	1.070 (0.611–1.877)	0.812
Tumor size (> 2 cm vs ≤ 2 cm)	0.886 (0.553–1.419)	0.614	1.391 (0.775–2.499)	0.269
LN (positive vs negative)	3.331 (2.015–5.505)	< 0.001	2.873 (1.566–5.270)	0.001
FFAR4 (high vs low)	**2.183 (1.360–3.504)**	**0.001**	**2.102 (1.173–3.766)**	**0.013**
Age at diagnosis	0.987 (0.966–1.008)	0.209	0.999 (0.974–1.024)	0.940
Grade (3 vs 2 or 1)	1.777 (1.136–2.780)	0.012	1.122 (0.646–1.950)	0.682
Tumor size (> 2 cm vs ≤ 2 cm)	0.934 (0.585–1.491)	0.776	1.418 (0.791–2.541)	0.241
LN (positive vs negative)	3.011 (1.840–4.929)	< 0.001	2.644 (1.454–4.808)	0.001

Abbrevation: *LN* Lymph node status, *HR* hazard ratio, *CI* confidence interval

FFAR4 H-score as a continuous variable (0 to 300, increment in every 10 point) or as a binary variable (high vs low). Age in years as a continuous variable. All Wald statistical tests were two-sided; boldface was used to indicate the key variable (FFAR4)

Interestingly, in HRPBC patients, FFAR4 expression was associated with higher Ki-67 index (*P* = 0.008) and to a lesser extent, worse histological grade (*P* = 0.087) (Table 1), suggesting that higher FFAR4 expression in HRPBC patients might be associated with more aggressive breast cancer phenotypes in HRPBC.

### Prognostic value of FFAR4 in validation datasets

**Fig. 4:**
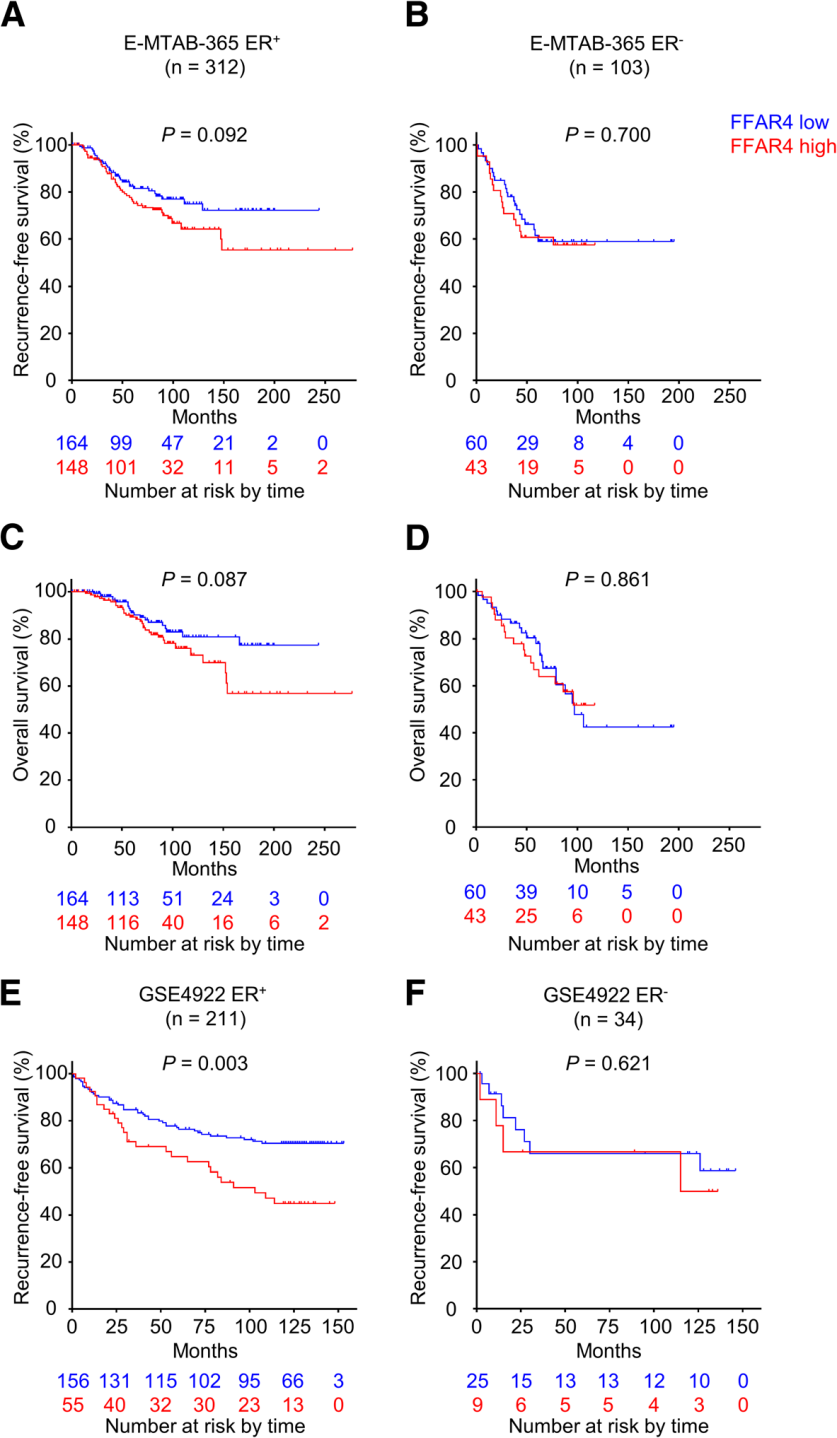
FFAR4’s prognostic value in the validating datasets. Kaplan- Meier curves of recurrences based on FFAR4 expression in public datasets. (**a** and **c**) RFS and OS of the E-MTAB-365 ER-positive cohort (*n* = 312). (**b** and **d**) RFS and OS of the E-MTAB-365 ER-negative cohort (*n* = 103). **e** RFS of the GSE4922 ER-positive cohort (*n* = 211). **f** RFS of the GSE4922 ER-negative cohort (*n* = 34). Bottom numbers indicate patients at risk at different time points

To validate our findings, two online breast cancer microarray datasets E-MTAB-365 and GSE4922 were employed and stratified into ER-positive cohorts and ER-negative cohorts. FFAR4 mRNA levels were extracted and patients were labeled accordingly. In the E-MTAB-365 ER-positive cohort, 74 patients (23.7%) developed disease recurrences, patients with higher FFAR4 mRNA levels showed worse RFS (15-year RFS; FFAR4-low vs. high: 72.2% vs. 55.3%; *P* = 0.092; Fig. 4a) and overall survival (OS, 15-year OS; FFAR4-low vs. high: 77.4% vs. 56.8%; *P* = 0.087; Fig. 4c) compared with FFAR4-low patients, though not reaching significant level, possibly due to the relatively less events recorded or differences in FFAR4 detection methods. In the GSE4922 ER-positive cohort, 76 patients (36.0%) developed disease recurrences, patients with higher FFAR4 mRNA levels showed worse RFS (10-year RFS; FFAR4-low vs. high: 68.6% vs. 43.3%; *P* = 0.003; Fig. 4e) compared with FFAR4-low patients. In contrast, FFAR4 was not associated with survival in ER-negative cohorts (Fig. 4b, d and f). In multivariable analysis, FFAR4 remained prognostic for RFS (E-MTAB-365: continuous, HR: 1.168, *P* = 0.070; binary, HR: 1.512, *P* = 0.086; GSE4922: continuous, HR 1.236, *P* = 0.033; binary, HR: 1.738, *P* = 0.026. Table 3). The survival analysis results from the validation datasets indicate that FFAR4’s prognostic value is different between ER-positive and ER-negative cohorts, which is consistent with our findings in the exploration cohort.

**Table 3:** Multivariable analyses in validation ER-positive cohorts

Cohort	Covariates	Recurrence-free survival	
		HR (95% CI)	*P*
E-MTAB-365 ER-positive cohort^a^			
	**FFAR4 (continuous)**	**1.168 (0.988–1.381)**	**0.070**
	Age at diagnosis	1.015 (0.995–1.037)	0.149
	Grade (3 vs 2 or 1)	1.656 (0.995–2.756)	0.052
	LN (positive vs negative)	1.687 (0.986–2.888)	0.056
	**FFAR4 (high vs low)**	**1.512 (0.944–2.421)**	**0.086**
	Age at diagnosis	1.014 (0.993–1.035)	0.183
	Grade (3 vs 2 or 1)	1.660 (0.998–2.759)	0.051
	LN (positive vs negative)	1.659 (0.968–2.843)	0.066
GSE4922 ER-positive cohort			
	**FFAR4 (continuous)**	**1.236 (1.017–1.507)**	**0.033**
	Age at diagnosis	1.003 (0.985–1.021)	0.723
	Grade (3 vs 2 or 1)	1.404 (0.792–2.489)	0.245
	Tumor size (> 2 cm vs ≤ 2 cm)	2.215 (1.349–3.639)	0.002
	LN (positive vs negative)	1.542 (0.945–2.517)	0.083
	**FFAR4 (high vs low)**	**1.738 (1.068–2.828)**	**0.026**
	Age at diagnosis	1.002 (0.984–1.020)	0.834
	Grade (3 vs 2 or 1)	1.308 (0.728–2.350)	0.370
	Tumor size (> 2 cm vs ≤ 2 cm)	2.213 (1.347–3.635)	0.002
	LN (positive vs negative)	1.551 (0.952–2.527)	0.078

Abbrevation: *LN* Lymph node status, *HR* hazard ratio, *CI* confidence interval

FFAR4 mRNA level as a continuous variable or as a binary variable (high vs low)

^a^The E-MTAB-365 cohort contains no tumor size information. All Wald statistical tests were two-sided; boldface was used to indicate the key variable (FFAR4)

### FFAR4 signaling attenuates tamoxifen efficacy

The clinical data indicated that FFAR4’s prognostic value resides predominantly in hormone receptor-positive patients, who were uniformly subjected to anti-hormone adjuvant therapy (tamoxifen), which hormone receptor-negative patients had not received [41, 42]. Therefore, we hypothesized that FFAR4 may interfere with tamoxifen’s therapeutic effect.

**Fig. 5:**
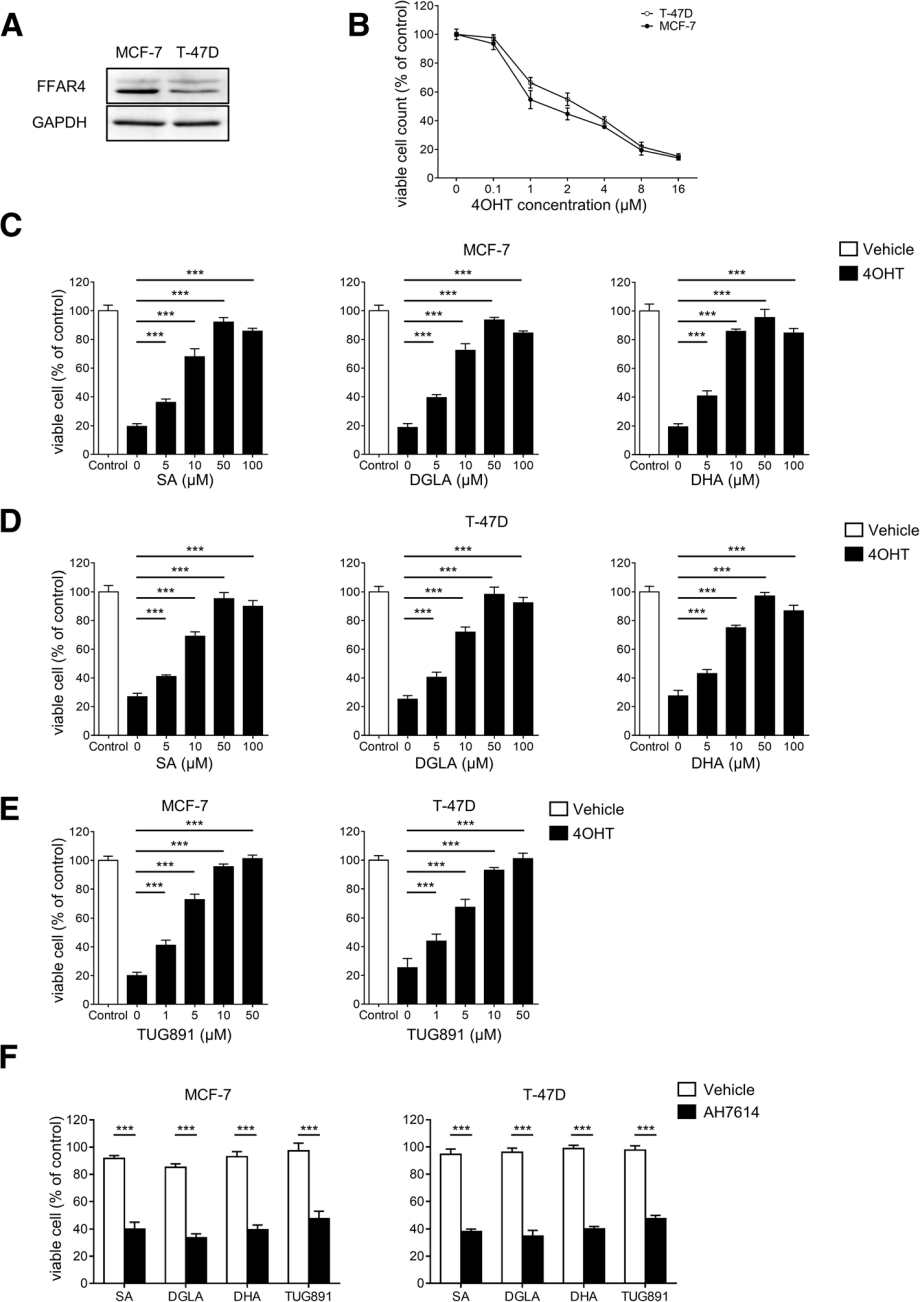
FFAR4 activation via endogenous or synthetic ligands confers tamoxifen resistance in HRPBC. **a** Immunoblot analyses of FFAR4 expression in human HRPBC cell lines MCF-7 and T-47D. **b** Cell viability of MCF-7 and T-47D cells treated with different concentrations of 4OHT (0, 0.1, 1, 2, 4, 8 and 16 μM). Cell viability of MCF-7 (**c**) and T-47D (**d**) cells treated with 4OHT (8 μM) and different concentrations (0, 5, 10, 50 and 100 μM) of endogenous FFAR4 ligands SA, DGLA and DHA. **e** Cell viability of MCF-7 and T-47D cells treated with 4OHT (8 μM) and different concentrations (0, 1, 5, 10 and 50 μM) of synthetic FFAR4 ligand TUG891. **f** Cell viability of MCF-7 and T-47D cells treated with 4OHT (8 μM) and TUG891 (10 μM) with or without AH7614 (10 μM) preincubation (30 min). Cells were seeded in 96-well plate, allowed to grow for 48 h before treatments with indicated reagents for 48 h. Cell viabilities were then measured using WST-1 substrates. Data were representative of three independent experiments. 4OHT, 4-hydroxytamoxifen. Error bars represent standard deviation of cell viability (%). *** *P* <  0.001

To validate this hypothesis, FFAR4 expression was evaluated in human HRPBC cell lines MCF-7 and T-47D by immunoblotting (Fig. 5a). To minimize disturbance of serum FFAR4 ligands and provide stable estrogen concentration in the culture system, cells were plated in phenol red-free medium supplemented with heat-inactivated charcoal-stripped serum and 1 nM β-estradiol for 48 h before and during any treatment. HRPBC cells were treated with different concentrations of active tamoxifen metabolite 4OHT [43], and 8 μM was chosen as optimal dose for subsequent experiments (Fig. 5b). We next investigated that whether breast cancer tissue-enriched endogenous FFAR4 ligands (SA, DGLA, DHA) affect tamoxifen’s therapeutic effect in HRPBC cells. Intriguingly, a significant increase in cell viability was observed in all 3 endogenous FFAR4 ligand-treated groups compared with vehicle-treated groups (Fig. 5c-d). These results indicate that endogenous FFAR4 ligands in breast cancer tissues can indeed interfere with tamoxifen’s efficacy.

**Fig. 6:**
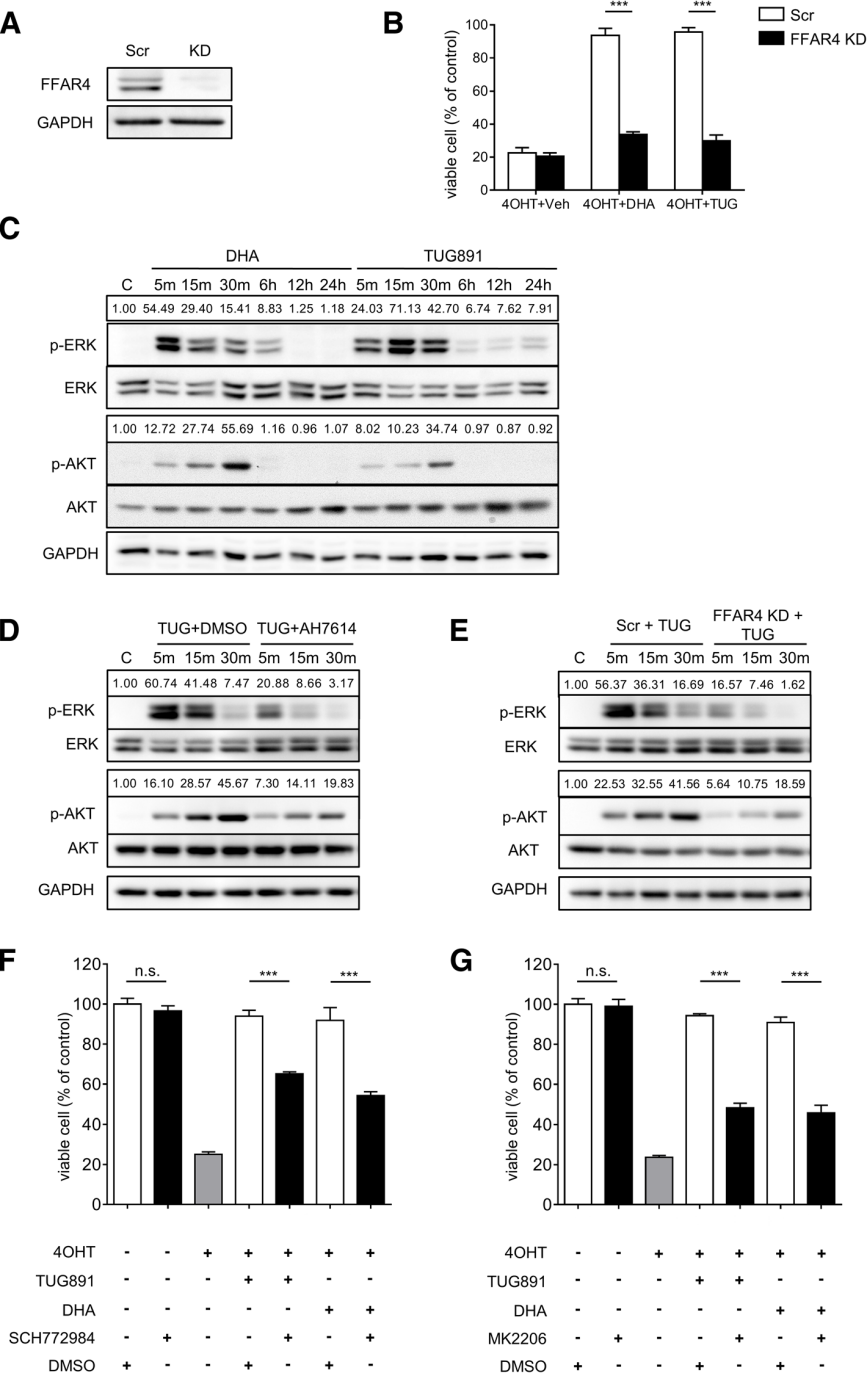
FFAR4-induced tamoxifen resistance is dependent on ERK and AKT pathways. **a** MCF-7 cells were transfected with scramble shRNA (Scr) or FFAR4 shRNA (FFAR4 KD) expressing constructs. **b** Cell viability of MCF-7 Scr/FFAR4 KD cells treated with 4OHT (8 μM) combined with DHA (50 μM), TUG891 (10 μM) or vehicle. **c** MCF-7 cells were seeded in 6-well plates for 48 h then treated with DHA (100 μM) or TUG891 (10 μM); **d** MCF-7 cells were seeded in 6-well plates for 48 h then treated with TUG891 (10 μM) combined with vehicle or AH7614 (10 μM); **e** MCF-7 Scr or FFAR4 KD cells were seeded in 6-well plates for 48 h then treated with TUG891 (10 μM); **c**-**e** cells were seeded in 6-well plates for 48 h then treated for the indicated time; phospho-ERK, ERK, phospho-AKT, AKT were measured by immunoblotting, the numbers above the lanes indicate the ratio of pERK/ERK and pAKT/AKT. GAPDH was used as loading control. **f** and **g** MCF-7 cells were seeded in 96-well plates for 48 h, then treated with 4OHT (8 μM) combined with DHA (50 μM), TUG891 (10 μM) or vehicle for 48 h, ERK1/2 inhibitor SCH772984 (10 μM) (**f**) or AKT inhibitor MK2206 (5 μM) (**g**) were added 30 min prior to other reagents, cell viabilities were measured using WST-1 substrates. The results shown are representative of three independent experiments. 4OHT, 4-hydroxytamoxifen. Error bars represent standard deviation. n.s.: not significant, *** *P* <  0.001

To confirm FFAR4’s role in fatty acids-induced tamoxifen resistance, a synthetic FFAR4 agonist TUG891 [44] was employed, as expected, TUG891 also induced tamoxifen resistance (Fig. 5e). Moreover, a FFAR4 selective antagonist AH7614 [45] significantly restored tamoxifen’s efficacy under endogenous or synthetic FFAR4 ligands treatment (Fig. 5f). Furthermore, shRNA-mediated knockdown of FFAR4 significantly abrogated DHA and TUG891 induced tamoxifen resistance (Fig. 6a-b). These results confirmed FFAR4 as the convict for the abrogation of tamoxifen’s efficacy.

### FFAR4 signaling mediates tamoxifen resistance via ERK and AKT pathways

As demonstrated above, FFAR4 signaling, activated by endogenous or synthetic FFAR4 ligands, confers tamoxifen resistance in HRPBC, but the underlying signaling events remained unknown. Previous studies have shown that FFAR4 activates ERK and AKT pathways [10, 15, 17, 20, 46], and both pathways lead to tamoxifen resistance [47–51]. To delineate the cellular signaling events involved in FFAR4 conferred tamoxifen resistance, the phosphorylation of ERK and AKT was detected by immunoblotting at different time points after FFAR4 stimulation. Consistent with previous reports, both DHA and TUG891 induced rapid ERK and AKT phosphorylation (Fig. 6c). This effect was blocked by AH7614 pretreatment or FFAR4 knockdown (Fig. 6d-e). Additionally, both ERK and AKT inhibition significantly abolished FFAR4-induced tamoxifen resistance, while the inhibitors alone did not affect cell viability at applied doses (Fig. 6f-g). These results indicate that FFAR4-induced tamoxifen resistance is dependent on the activation of ERK and AKT pathways.

The in vitro results validated our hypothesis that FFAR4 can dampen tamoxifen’s efficacy, mechanistically explaining our clinical finding that FFAR4 plays a fundamental role in tamoxifen-treated HRPBC patients.

## Discussion

FFAR4 is a receptor for long-chain free fatty acids and the most recent member of the fatty acid receptor family. Past research interests have been focused on its proposed effects in regulating inflammation and metabolism related diseases [15, 52]. We recently demonstrated that FFAR4 possesses tumor promoting properties in adipose tissues-adjacent malignancies like colorectal carcinoma [20]. Yet studies addressing its clinical significance remain lacking. In this report, we showed that FFAR4 is aberrantly expressed in human breast cancer, and identified several endogenous FFAR4 ligands with elevated proportions in breast cancer tissues. We showed in the exploration cohort and in two validation datasets that FFAR4 is prognostic in tamoxifen-treated HRPBC patients but not so in hormone receptor-negative patients. Furthermore, FFAR4 signaling, activated by endogenous or synthetic ligands, was shown to dampen tamoxifen efficacy in HRPBC cells. Considering all the evidence collected, we conclude that FFAR4 confers tamoxifen resistance in HRPBC.

To our best knowledge, this is the first report revealing FFAR4’s clinical utility in breast cancer. We first analyzed FFAR4 expression by IHC in 307 breast cancer cases in the exploration cohort. Aberrant FFAR4 expression was detected in the breast cancer tissues while normal breast epithelial cells remained negative, indicating FFAR4 may play a role in breast cancer biology.

Besides FFAR4 expression, FFAR4 ligand distribution in situ is also of great concern, as previous investigations on fatty acid composition in human breast tissues didn’t include breast cancer tissues [21–23], and none of these studies were conducted in Chinese cohorts. Using a GC-MS approach, we examined the fatty acids in cancerous and normal breast tissues and identified 24 long-chain fatty acids, with significantly different FFAR4 ligand distribution. The total fatty acid amount was significantly lower in cancerous tissues, which was expected since adipocytes were either replaced by cancer cells or lost bulk amounts of triglycerides from lipolysis during breast cancer progression. The dramatic decrease of palmitic acid (C16:0), oleic acid (C18:1n-9) and linoleic acid (C18:2n-6), major fatty acids composing human triglycerides [53, 54], further supported this notion. Although these 3 fatty acids were confirmed FFAR4 ligands, they were unable to contribute to FFAR4 agonism when stored in adipocytes in the form of fatty acid esters composing triglycerides, as the carboxyl group is indispensable for FFAR4 ligand stimulation [10]. Despite the decrease of triglyceride fatty acids, five FFAR4 ligands were enriched in breast cancer tissues, which were found to induce tamoxifen resistance on HRPBC cells. The FFAR4 ligands elevated proportions remained the same between hormone receptor-positive and negative cases, indicating that the FFAR4 ligand distributions are not likely regulated by hormone receptors in breast cancer tissues.

We also found elevated proportions or quantities of other long-chain fatty acids in breast cancer tissues with previously unconfirmed FFAR4 affinity (e.g. C20:4n-6, arachidonic acid, AA). Interestingly, a very recent study showed AA is also a FFAR4 ligand [55], and it showed elevated quantity and proportion in breast cancer tissues in our GC-MS data. AA may be an important endogenous FFAR4 ligand in situ. It will be necessary to explore whether these fatty acids also function as FFAR4 ligands in future studies. The altered fatty acid profiles indicated that despite fatty acid expenditure, certain long-chain fatty acids were preserved, or de novo synthesized during breast tumorigenesis and progression, and thus may serve as ligands for FFAR4 and affect breast cancer biology and patient outcome.

With FFAR4 and its endogenous ligands in breast cancer tissues identified, we next examined its prognostic value in patients. In the exploration cohort, FFAR4 was demonstrated independently prognostic for HRPBC patients, while there was no significant association between FFAR4 expression and outcome in hormone receptor-negative patients.

Similar contrasting results between ER-positive and ER-negative patients were also observed in two validation datasets. Albeit the distinct pattern of FFAR4’s prognostic value regarding different ER status, statistical significance was reached in the GSE4922 ER-positive cohort, but not the E-MTAB-365 ER-positive cohort (*P* = 0.092). There are two possible explanations: First, the E-MTAB-365 ER-positive sub-cohort has relatively lower relapse rate compared to the exploration HRPBC cohort and GSE4922 ER-positive cohort (23.7% in E-MTAB-365 vs. 38.7% in exploration HRPBC and 36.0% in GSE4922) which may have hindered the discerning power of survival analysis; Second, in microarray datasets, gene expressions were analyzed using total tissue mRNA, and mRNA from cell types other than breast cancer cells may interfere with the expression of certain genes. In the case of FFAR4, it is known that FFAR4 is also expressed in adipocytes and macrophages, both cell types are abundantly present in breast cancer tissues and may affect FFAR4’s expression read-out in microarray datasets. On the other hand, pathologists can easily rule out FFAR4 staining on cell types other than breast cancer cells in IHC-stained sections.

A recent literature reviewed the current status of prognostic molecular biomarkers in breast cancer [56]. In this review, the authors classified the biomarkers into highly reliable markers (HR ≥ 2) and low/moderately reliable markers (HR < 2) according to reported risk ratios. Under this criteria, FFAR4 IHC H-score qualifies as highly reliable marker, but not the FFAR4 microarray mRNA expression. This might be due to the superior discerning ability in IHC technique (tumor cell expression only) over microarray (total tissue RNA). A prospectively designed cohort using IHC method to detect FFAR4 expression and evaluate its prognostic value is warranted.

FFAR4 status as a determinant for recurrence and survival in HRPBC patients in concert with abundant presence of FFAR4 ligands in breast cancer tissues provides novel insights into the importance of FFAR4 signaling in breast cancer biology, and prompted us to investigate the possible mechanism of FFAR4 signaling behind the phenomena. The clinical data indicated that FFAR4’s prognostic value mainly applies to HRPBC patients, while there was no association with disease outcome in hormone receptor-negative patients. A major difference between hormone receptor-positive and hormone receptor-negative patients is that HRPBC patients, which composes about 70% of all breast cancer cases, uniformly receive tamoxifen as standard adjuvant anti-hormone therapy, which hormone receptor-negative patients do not receive [41, 42]. Therefore, it’s plausible that FFAR4 affects HRPBC patients’ outcome via interfering tamoxifen’s therapeutic effect. Here, we showed that FFAR4 is expressed in HRPBC cells, endogenous and synthetic FFAR4 ligand treatment significantly dampened tamoxifen’s efficacy in HRPBC cells, and FFAR4 knockdown or antagonist treatment significantly abrogated this effect, therewith confirming that FFAR4 plays a crucial role in tamoxifen resistance.

Our results also delineated the molecular mechanism underlying FFAR4-induced tamoxifen resistance in HRPBC. Previous studies have shown that FFAR4 mediated ERK phosphorylation via coupling G_q/11_ family proteins, and AKT phosphorylation via recruiting arrestins [10, 15, 17, 20, 44, 46], and it has been reported that both ERK and AKT activation leads to tamoxifen resistance by ligand-independent phosphorylation of estrogen receptor [47–51]. In this study, we found that both endogenous and synthetic FFAR4 ligand treatment induced rapid ERK1/2 and AKT phosphorylation in HRPBC cells. Moreover, suppression of either ERK or AKT signaling abrogated FFAR4-induced tamoxifen resistance, suggesting that FFAR4-mediated tamoxifen resistance in HRPBC is dependent on both ERK and AKT pathways.

It is reported that one class of FFAR4 ligands, the ω-3 polyunsaturated fatty acids (ω-3 PUFA, e.g. DHA and eicosapentaenoic acid), exert anti-tumor properties [57–60]. These reports seem contradictory with our findings, and we would like to clarify. ω-3 PUFA exerted effects can be classified as FFAR4-dependent and FFAR4-independent, the ω-3 PUFA’s suppressive effects on breast cancer is FFAR4-independent, as Chung and colleagues demonstrated using a FFAR4-knockout model [61]. Furthermore, some of the studies employed ω-3 PUFA esters [57, 62, 63], which cannot activate FFAR4 as they lack the carboxyl group, an indispensable structure for FFAR4 stimulation [10], yet observed anti-cancer effects nonetheless. These data indicated that targets other than FFAR4 are responsible for ω-3 PUFA’s anti-tumor effects. It is worth noting that regular instead of fatty acid-stripped serum was used in these literatures, the actual ω-3 PUFA concentrations might be higher than the researchers intended, resulting in unspecific/off-target cytotoxic effects. Collectively, ω-3 PUFA may confer anti-tumor effects, but these effects are not mediated via FFAR4.

Of note, future studies are needed to address several aspects not covered by our report. First, since FFAR4 is associated with higher Ki-67 expression and worse histological grade in HRPBC patients, it may be associated with an intrinsically more aggressive phenotype within the HRPBC cohort, which may also contribute to a worse prognosis under unknown mechanisms other than FFAR4-induced tamoxifen resistance reported in this study. To validate this possibility, HRPBC cohorts without tamoxifen treatment are required in following studies. Second, given FFAR4’s negative impact on breast cancer prognosis, FFAR4 signaling pathway may provide novel targets for future anti-breast cancer therapies. A synthetic FFAR4 antagonist is already available for research purposes [45], and it is essential to perform further investigations to provide breast cancer patients with potential treatment options, especially in tamoxifen resistant HRPBC cases. Third, FFAR4 was also reported to possess tumor promoting properties in colorectal and pancreatic cancer [20, 64], and a recent genome-wide association study showed that a genetic variant of FFAR4 was associated with increased risk for lung cancer [65]. Further studies are mandatory to investigate potential prognostic value and molecular mechanisms of FFAR4 in these malignancies. Finally, considering the existence of FFAR4 in several human malignancies, we advise caution in the use of FFAR4 agonists or commercially available fish oil products for treatment of metabolic and inflammatory diseases in cancer patients due to systemic consequences.

## Conclusions

In conclusion, we have confirmed the aberrant expression of FFAR4 in human breast cancer patients and demonstrated that FFAR4 can risk-stratify tamoxifen-treated HRPBC patients. Elevated proportions of endogenous FFAR4 ligands were identified in human breast cancer compared with normal tissues. Both endogenous and synthetic FFAR4 ligands induced prominent tamoxifen resistance in HRPBC cells, which was dependent on ERK and AKT activation. These findings revealed an unexpected aspect of the complex interactions between cancer cells and tumor microenvironment - FFAR4 and its endogenous ligands - and provides us with mechanistic insights into the links between tissue lipid profile and cancer biology. Our results suggest a novel role of FFAR4 in breast cancer endocrine resistance and it may serve as a potential target for future therapeutic strategies.

## Additional files

Additional file 1: **Figure S1.** Cohort selection. Breast cancer cohort selection workflow visualized. **Figure S2.** Prognostic value of FFAR4 in different breast cancer subtypes. (PDF 139 kb)

Additional file 2: **Table S1.** Fatty Acid Quantities in Normal and Cancerous Breast Tissues. **Table S2.** Fatty Acid Proportions in Normal and Cancerous Breast Tissues. (PDF 195 kb)

## Abbreviations

BCSS: Breast cancer-specific survival
DGLA: Dihomo-gamma-linolenic acid
DHA: Docosahexaenoic acid
DPA: Docosapentaenoic acid
DTA: Docosatetraenoic acid
ER: Estrogen receptor
FFAR4: Free fatty acid receptor 4
FFPE: Formalin-fixed and paraffin-embedded
GC-MS: Gas chromatography-coupled mass spectrometry
HER2: Human epidermal growth factor receptor 2
HR: Hazard ratio
HRPBC: Hormone receptor-positive breast cancer
IHC: Immunohistochemistry
PR: Progesterone receptor
RFS: Recurrence-free survival
SA: Stearic acid
