# Supplementary material for: Aberrant fatty acid profile and FFAR4 signaling confer endocrine resistance in breast cancer
Source: J Exp Clin Cancer Res. 2019 Feb 22;38:100. doi: 10.1186/s13046-019-1040-3 (PMC6387561; doi:10.1186/s13046-019-1040-3)

Supplemental Figure Legends.

Supplemental Figure 1. Cohort selection. Breast cancer cohort selection workflow visualized.

Supplemental Figure 2. Prognostic value of FFAR4 in different breast cancer subtypes. Kaplan-Meier curves of patient outcomes based on FFAR4 expression. (A) RFS and (B) BCSS of Luminal A cohort (n = 75). (C) RFS and (D) BCSS of Luminal B HER2- cohort (n = 86). (E) RFS and (F) BCSS of Luminal B HER2+ cohort (n = 51). (G) RFS and (H) BCSS of HER2 non-luminal cohort (n = 41). (I) RFS and (J) BCSS of Triple negative cohort (n = 54). Bottom numbers indicate patient numbers at risk on different time points.

Supplemental Figure 1

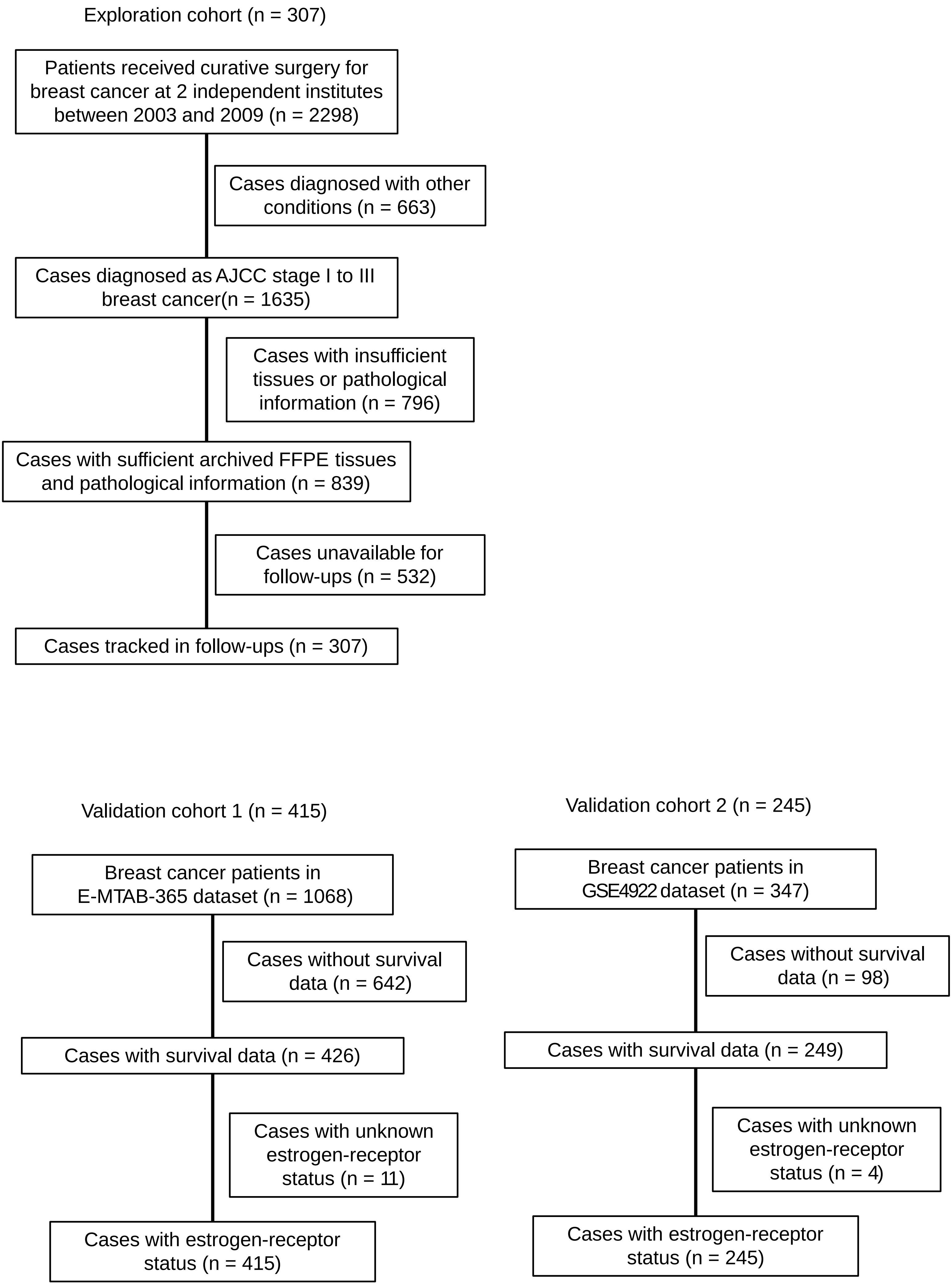

Supplemental Figure 2

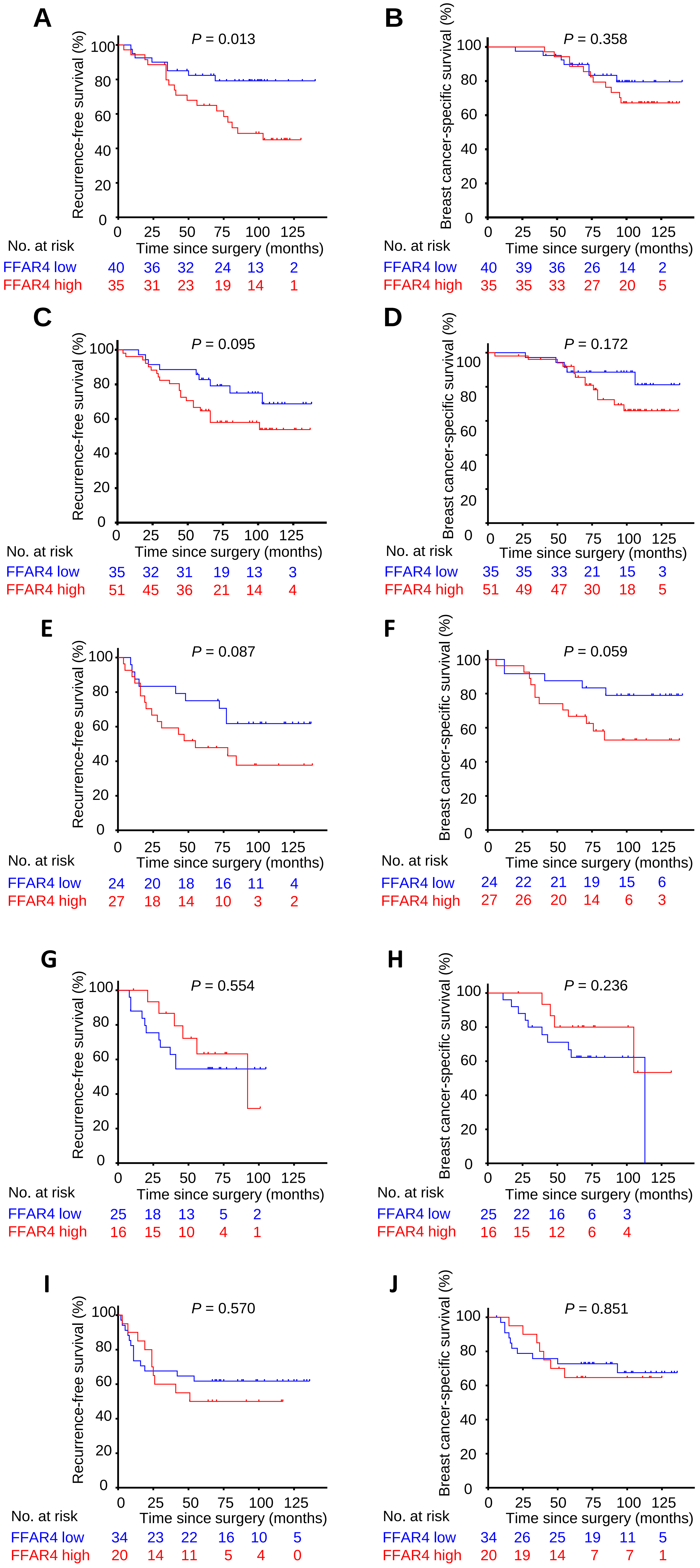

Supplement: Supplementary file 1 — Figure S1. Cohort selection. Breast cancer cohort selection workflow visualized. Figure S2. Prognostic value of FFAR4 in different breast cancer subtypes. (PDF 139 kb) [file 13046_2019_1040_MOESM1_ESM.pdf]
