# Supplementary material for: Aberrant fatty acid profile and FFAR4 signaling confer endocrine resistance in breast cancer
Source: J Exp Clin Cancer Res. 2019 Feb 22;38:100. doi: 10.1186/s13046-019-1040-3 (PMC6387561; doi:10.1186/s13046-019-1040-3)
Supplement: Supplementary file 2 — Table S1. Fatty Acid Quantities in Normal and Cancerous Breast Tissues. Table S2. Fatty Acid Proportions in Normal and Cancerous Breast Tissues. (PDF 195 kb) [file 13046_2019_1040_MOESM2_ESM.pdf]

Supplemental Table 1. Fatty Acid Quantities in Normal and Cancerous Breast Tissues

	**all patients**			**hormon receptor-positive**			**hormon receptor-negative**		
Fatty acid	Normal (n = 19)	Tumor (n = 19)	*P*	Normal (n = 10)	Tumor (n = 10)	*P*	Normal (n = 9)	Tumor (n = 9)	*P*
total fatty acid	**117.18 ± 86.27**	**28.03 ± 17.96**	< **0.001**	**82.85 ± 63.60**	**23.77 ± 8.10**	**0.017**	**155.32 ± 95.32**	**32.77 ± 24.58**	**0.003**
C12:0	0.12 ± 0.10	0.02 ± 0.02	< 0.001	0.11 ± 0.10	0.02 ± 0.01	0.021	0.14 ± 0.10	0.02 ± 0.02	0.005
C14:0*	1.21 ± 0.82	0.27 ± 0.15	< 0.001	0.87 ± 0.55	0.24 ± 0.10	0.007	1.58 ± 0.93	0.31 ± 0.19	0.002
C15:0	0.10 ± 0.06	0.05 ± 0.03	0.003	0.08 ± 0.05	0.04 ± 0.02	0.051	0.13 ± 0.07	0.06 ± 0.04	0.038
C16:0*	24.83 ± 18.94	5.79 ± 3.58	< 0.001	17.86 ± 13.71	4.99 ± 1.96	0.016	32.56 ± 21.63	6.67 ± 4.79	0.005
C16:1n-7*	3.36 ± 2.29	0.81 ± 0.51	< 0.001	2.81 ± 2.34	0.70 ± 0.34	0.005	3.98 ± 2.20	0.93 ± 0.66	0.001
C18:0*	**3.86 ± 3.01**	**2.16 ± 1.28**	**0.064**#	**2.76 ± 1.90**	**1.96 ± 0.40**	**0.216**#	**5.07 ± 3.62**	**2.38 ± 1.84**	**0.089**#
C18:1n-9*	44.08 ± 30.54	9.60 ± 7.51	< 0.001	29.36 ± 18.13	7.62 ± 2.79	0.004	60.44 ± 34.01	11.81 ± 10.39	0.002
C18:1n-7	0.36 ± 0.79	0.69 ± 0.97	0.126	0.15 ± 0.64	0.55 ± 0.57	0.073	0.59 ± 1.13	0.85 ± 1.30	0.767
C18:2n-6*	35.08 ± 31.46	5.74 ± 4.41	< 0.001	26.01 ± 27.78	5.02 ± 2.15	0.013	45.16 ± 33.79	6.53 ± 6.10	0.006
C18:3n-6*	**0.04 ± 0.03**	**0.03 ± 0.05**	**0.147**#	**0.04 ± 0.03**	**0.03 ± 0.05**	**0.285**#	**0.04 ± 0.03**	**0.03 ± 0.05**	**0.596**#
C20:0	0.10 ± 0.08	0.05 ± 0.03	0.014	0.07 ± 0.06	0.04 ± 0.01	0.074	0.13 ± 0.09	0.07 ± 0.04	0.090
C20:1n-9	0.78 ± 0.49	0.26 ± 0.16	< 0.001	0.54 ± 0.35	0.20 ± 0.07	0.021	1.04 ± 0.51	0.32 ± 0.21	0.002
C20:2n-6	0.53 ± 0.35	0.23 ± 0.12	< 0.001	0.39 ± 0.22	0.23 ± 0.10	0.079	0.68 ± 0.42	0.24 ± 0.14	0.006
C20:3n-6*	**0.33 ± 0.24**	**0.38 ± 0.25**	**0.453**#	**0.23 ± 0.15**	**0.41 ± 0.31**	**0.051**#	**0.44 ± 0.28**	**0.36 ± 0.19**	**0.440**#
C20:4n-6	0.67 ± 0.35	1.04 ± 0.48	< 0.001	0.54 ± 0.26	0.94 ± 0.30	< 0.001	0.81 ± 0.40	1.15 ± 0.62	0.086
C20:5n-3*	**0.04 ± 0.04**	**0.02 ± 0.03**	**0.243**#	**0.03 ± 0.03**	**0.02 ± 0.03**	**0.698**#	**0.05 ± 0.04**	**0.03 ± 0.03**	**0.254**#
C22:0	0.02 ± 0.02	0.04 ± 0.02	0.001	0.01 ± 0.02	0.03 ± 0.01	0.006	0.02 ± 0.03	0.06 ± 0.03	0.021
C22:1n-9	0.09 ± 0.10	0.05 ± 0.04	0.071	0.06 ± 0.08	0.03 ± 0.02	0.374	0.12 ± 0.11	0.07 ± 0.05	0.084
C22:4n-6*	**0.26 ± 0.20**	**0.22 ± 0.12**	**0.494**#	**0.19 ± 0.12**	**0.20 ± 0.08**	**0.571**#	**0.34 ± 0.23**	**0.24 ± 0.16**	**0.149**#
C22:5n-3*	0.20 ± 0.13	0.15 ± 0.09	0.037	**0.15 ± 0.08**	**0.13 ± 0.06**	**0.232**#	**0.26 ± 0.15**	**0.17 ± 0.11**	**0.092**#
C22:5n-6	0.04 ± 0.04	0.04 ± 0.04	0.777	0.04 ± 0.03	0.04 ± 0.04	0.503	0.04 ± 0.05	0.04 ± 0.04	0.933
C22:6n-3*	**0.24 ± 0.15**	**0.22 ± 0.11**	**0.621**#	**0.20 ± 0.13**	**0.21 ± 0.09**	**0.700**#	**0.28 ± 0.16**	**0.23 ± 0.13**	**0.370**#
C24:0	0.01 ± 0.01	0.06 ± 0.04	< 0.001	0.01 ± 0.01	0.04 ± 0.02	0.001	0.01 ± 0.01	0.08 ± 0.02	0.006
C24:1n-9	0.02 ± 0.03	0.09 ± 0.07	< 0.001	0.01 ± 0.02	0.07 ± 0.03	< 0.001	0.03 ± 0.04	0.13 ± 0.08	0.003
Total n-3 PUFA	0.48 ± 0.29	0.39 ± 0.20	0.179	0.38 ± 0.23	0.36 ± 0.17	0.777	0.59 ± 0.33	0.43 ± 0.23	0.169
Total n-6 PUFA	36.95 ± 32.31	7.69 ± 5.09	< 0.001	27.43 ± 28.18	6.88 ± 2.51	0.013	47.52 ± 34.89	8.58 ± 7.03	0.007
Total MUFA	48.68 ± 33.04	11.51 ± 8.38	< 0.001	32.92 ± 20.11	9.17 ± 3.20	0.005	66.19 ± 36.68	14.10 ± 11.49	0.002
Total SFA	30.24 ± 22.80	8.45 ± 4.66	< 0.001	21.78 ± 16.28	7.37 ± 2.45	0.022	39.64 ± 26.13	9.65 ± 6.24	0.007

Abbrevation: PUFA = polyunsaturated fatty acid; MUFA = monounsaturated fatty acid; SFA = saturated fatty acid; Values show as mean ± standard deviation.
Fatty acids are shown as fatty acid(μg)/tissue(mg). *FFAR4 ligands. #FFAR4 ligands with unabated quantity in tumor tissues.

Supplemental Table 2. Fatty Acid Proportions in Normal and Cancerous Breast Tissues

Fatty acid	all patients			hormon receptor-positive			hormon receptor-negative		
	Normal (n = 19)	Tumor (n = 19)	*P*	Normal (n = 10)	Tumor (n = 10)	*P*	Normal (n = 9)	Tumor (n = 9)	*P*
C12:0	0.11 ± 0.04	0.08 ± 0.04	< 0.001	0.12 ± 0.05	0.09 ± 0.04	0.008	0.09 ± 0.03	0.06 ± 0.04	0.015
C14:0*	1.11 ± 0.30	1.00 ± 0.28	0.122	1.19 ± 0.37	1.00 ± 0.24	0.066	1.03 ± 0.17	1.00 ± 0.33	0.797
C15:0	0.10 ± 0.02	0.21 ± 0.16	0.001	0.11 ± 0.02	0.19 ± 0.09	0.024	0.09 ± 0.02	0.24 ± 0.21	0.011
C16:0*	20.99 ± 1.92	20.98 ± 2.74	0.992	21.36 ± 2.05	20.71 ± 1.29	0.283	20.58 ± 1.79	21.28 ± 3.86	0.614
C16:1n-7*	3.20 ± 1.24	2.96 ± 1.22	0.444	3.63 ± 1.51	2.90 ± 1.00	0.091	2.72 ± 0.63	3.02 ± 1.49	0.517
C18:0*	**3.56 ± 1.18**	**8.53 ± 3.37**	**< 0.001**#	**3.77 ± 1.27**	**8.57 ± 1.44**	**< 0.001**#	**3.33 ± 1.10**	**8.49 ± 4.81**	**0.009**#
C18:1n-9*	38.55 ± 6.07	31.67 ± 5.69	0.003	37.46 ± 4.83	31.70 ± 2.48	0.014	39.76 ± 7.32	31.63 ± 8.12	0.058
C18:1n-7	0.37 ± 0.64	2.56 ± 2.80	< 0.001	0.37 ± 0.74	2.40 ± 2.30	0.023	0.37 ± 0.55	2.74 ± 3.41	0.015
C18:2n-6*	28.17 ± 7.24	20.00 ± 4.13	< 0.001	28.13 ± 6.67	20.63 ± 3.85	0.006	28.21 ± 8.25	19.21 ± 4.53	0.003
C18:3n-6*	0.04 ± 0.02	0.14 ± 0.29	0.445	0.05 ± 0.02	0.12 ± 0.22	0.508	0.04 ± 0.02	0.16 ± 0.37	0.374
C20:0	0.09 ± 0.03	0.21 ± 0.13	< 0.001	0.10 ± 0.04	0.16 ± 0.04	0.001	0.09 ± 0.02	0.26 ± 0.17	0.008
C20:1n-9	0.74 ± 0.30	1.00 ± 0.46	0.004	0.72 ± 0.22	0.90 ± 0.28	0.052	0.76 ± 0.38	1.11 ± 0.60	0.038
C20:2n-6	0.48 ± 0.12	0.91 ± 0.23	< 0.001	0.51 ± 0.13	0.97 ± 0.19	< 0.001	0.45 ± 0.10	0.84 ± 0.26	0.008
C20:3n-6*	**0.30 ± 0.11**	**1.49 ± 0.84**	**< 0.001**#	**0.31 ± 0.15**	**1.63 ± 0.94**	**0.001**#	**0.29 ± 0.07**	**1.33 ± 0.75**	**0.008**#
C20:4n-6	0.76 ± 0.35	4.38 ± 2.34	< 0.001	0.83 ± 0.29	4.19 ± 1.80	< 0.001	0.68 ± 0.40	4.58 ± 2.94	0.002
C20:5n-3*	0.04 ± 0.03	0.12 ± 0.19	0.063	0.03 ± 0.03	0.09 ± 0.14	0.398	0.05 ± 0.03	0.11 ± 0.14	0.11
C22:0	0.03 ± 0.04	0.20 ± 0.14	< 0.001	0.03 ± 0.05	0.15 ± 0.05	0.001	0.02 ± 0.03	0.25 ± 0.19	0.004
C22:1n-9	0.10 ± 0.11	0.21 ± 0.21	< 0.001	0.08 ± 0.08	0.13 ± 0.10	0.003	0.11 ± 0.14	0.29 ± 0.28	0.016
C22:4n-6*	**0.25 ± 0.10**	**0.85 ± 0.29**	**< 0.001**#	**0.26 ± 0.12**	**0.88 ± 0.34**	**< 0.001**#	**0.24 ± 0.08**	**0.81 ± 0.23**	**< 0.001**#
C22:5n-3*	**0.20 ± 0.08**	**0.55 ± 0.20**	**< 0.001**#	**0.21 ± 0.07**	**0.53 ± 0.25**	**0.002**#	**0.20 ± 0.08**	**0.57 ± 0.14**	**< 0.001**#
C22:5n-6	0.06 ± 0.08	0.14 ± 0.10	0.004	0.09 ± 0.10	0.16 ± 0.10	0.086	0.03 ± 0.04	0.12 ± 0.09	0.021
C22:6n-3*	**0.22 ± 0.08**	**0.85 ± 0.35**	**< 0.001**#	**0.24 ± 0.08**	**0.88 ± 0.44**	**0.001**#	**0.20 ± 0.07**	**0.81 ± 0.23**	**< 0.001**#
C24:0	0.01 ± 0.02	0.26 ± 0.31	< 0.001	0.02 ± 0.03	0.18 ± 0.09	0.001	0.01 ± 0.01	0.34 ± 0.44	0.008
C24:1n-9	0.02 ± 0.04	0.40 ± 0.33	< 0.001	0.02 ± 0.04	0.29 ± 0.16	< 0.001	0.03 ± 0.04	0.51 ± 0.43	0.008
Total n-3 PUFA	0.47 ± 0.16	1.54 ± 0.65	< 0.001	0.48 ± 0.15	1.51 ± 0.79	0.002	0.45 ± 0.17	1.58 ± 0.52	< 0.001
Total n-6 PUFA	30.22 ± 7.11	27.40 ± 5.80	0.171	30.27 ± 6.30	28.75 ± 2.57	0.721	30.16 ± 8.32	25.90 ± 7.97	0.096
Total MUFA	43.18 ± 6.76	39.39 ± 7.19	0.039	42.38 ± 5.39	38.51 ± 2.34	0.047	44.08 ± 8.27	40.36 ± 10.40	0.284
Total SFA	26.03 ± 2.38	31.57 ± 3.66	< 0.001	26.71 ± 2.30	31.11 ± 1.55	< 0.001	25.27 ± 2.34	32.08 ± 5.18	0.008

Abbrevation: PUFA = polyunsaturated fatty acid; MUFA = monounsaturated fatty acid; SFA = saturated fatty acid; Values show as mean ± standard deviation.
Fatty acids are shown as percentage of total fatty acids in tissue. *FFAR4 ligands. #FFAR4 ligands with elevated proportion in tumor tissues.
